# New insights into the regulation of TGF‐β/Smad and MPK signaling pathway gene expressions by nasal allergen and methacholine challenge test in asthma

**DOI:** 10.1002/clt2.12172

**Published:** 2022-07-02

**Authors:** Michał Gabriel Panek, Michał Seweryn Karbownik, Karol Maksymilian Górski, Marcelina Koćwin, Grzegorz Kardas, Mateusz Marynowski, Piotr Kuna

**Affiliations:** ^1^ Department of Internal Medicine, Asthma and Allergy Medical University of Lodz Lodz Poland; ^2^ Department of Pharmacology and Toxicology Medical University of Lodz Lodz Poland; ^3^ Polish Mother's Memorial Hospital Research Institute Lodz Poland

**Keywords:** asthma, immunology, inflammation, intranasal allergen and bronchial methacholine challenge, molecular allergy, Smad, TGF

## Abstract

**Background:**

Asthma is a heterogeneous chronic inflammatory disease of the bronchi, the course of which is significantly influenced by extrinsic factors (specific and non‐specific).

**Methods:**

The aim of this study was to evaluate the effect of these factors represented by nasal allergen challenge (specific factors) and methacholine challenge test (non‐specific) on changes in mRNA expression of genes encoding the TGF‐β (TGF‐β1 and TGF‐β3)‒Smad (mitogen‐activated protein kinase 1/3 [MPK1/3], Smad1/3/6/7) signaling pathway in asthmatic patients.

**Results:**

Seventy‐five subjects were included in the study, of whom 27 were applied an intranasal allergen provocation and 48 a methacholine provocation. There were 9 men and 18 women in the intranasal provocation group, and 17 men and 31 women in the methacholine test group. We found that both examined the types of challenges contributed to changes in the relative expression of genes of the TGF‐β (TGF‐β1 and TGF‐β3)‒Smad (MPK1/3, Smad1/3/6/7) signaling pathway in asthmatic patients. A decrease was noted for MAPK1, MAPK3, Smad3, Smad6, and Smad7 genes and an increase of up to 2.5 times for TGF‐β1 gene.

**Conclusions:**

Our experiment allows us to conclude that the change in the mRNA expression of the TGF‐β1–MPK1/3 and Smad3/6/7 genes occurs after an intranasal allergen and bronchial methacholine challenge.

## 1 INTRODUCTION

Asthma is a heterogeneous chronic inflammatory disease of the bronchi, the course of which is significantly influenced by extrinsic factors (specific and non‐specific).^1^, ^2^, ^3^ It should be pointed out that irrespective of the type of inducer of inflammation in asthma (specific factors, e.g.: allergens; non‐specific factors, e.g.: pollutants, or other analogous/related substances) bronchial remodeling occurs as a result of TGF‐β (Transforming Growth Factor beta) overexpression.^4^, ^5^, ^6^ It is a profibrotic cytokine that is found in humans in three isoforms (TGFβ‐1, TGFβ‐2, TGFβ‐3). It stimulates the process of growth and differentiation of many cell types, controls their proliferation and apoptosis, and stimulates fibroblasts and bronchial smooth muscle cells to control the metabolism of extracellular matrix (ECM) proteins. Production of TGF‐β is associated with the presence of eosinophils in the airways of asthmatic patients. Eosinophilic granulocytes secrete some other profibrogenic molecules, such as eosinophil cationic protein (ECP). The TGF‐β superfamily are considered to be a group of key mediators, playing a role in the regulation of allergic and non‐allergic inflammation. It has a significant impact on airway remodeling in asthma.^7^, ^8^, ^9^

Intracellular effectors of TGF‐β signaling include among others: Smad proteins (Mothers Against Decapentaplegic, *MAD* and *SMA* gene; the name is a combination of the names of two homologous proteins Sma and MAD found in *Caenorhabditis elegans* and *Drosophila melanogaster*). They are activated by TGF‐β receptors and travel to the cell nucleus, where they regulate the transcription of over 500 genes, including those responsible for bronchial remodeling.^10^, ^11^, ^12^, ^13^, ^14^, ^15^, ^16^, ^17^

TGF‐β cytokine activates the TGFβRI/TGFβRII receptor Activin Receptor‐Like Kinase‐5 (ALK5), and thereby induces intracellular stimulation of Mitogen‐Activated Protein Kinase 1 and 3 (MAPK), Transforming growth factor beta‐Activated Kinase 1 (TAK1), c‐Jun N‐terminal kinase (JNK), Extracellular signal‐Regulated Kinase (ERK) and p38 synthesis without the involvement of Smad proteins. The TGFβRI/TGFβRII receptor (ALK1) activates Smad1 in the Smad1/5 complex and in cooperation with Smad4 protein, activates the intranuclear Smad1/4/5 complex and Transcription Factors (TFs), leading to protein synthesis of the target genes. In turn, Smad6 and Smad7 proteins, which belong to the group of proteins that inhibit the TGF‐β‐Smad intracellular signaling pathway, attenuate the activation of ALK5 and ALK1 receptor signaling. The activation of TGFβRI/TGFβRII (ALK5) and TGFβRI/TGFβRII (ALK1) receptors is different and depends on the activation inducing factor: specific factors and non‐specific factors. Hence, the cellular response of TGF‐β/Smad and mitogen‐activated protein kinase (MPK) signal pathway proteins to the nasal allergen challenge and methacholine challenge test is especially important in the pathogenesis of asthma, including bronchial remodeling.^5^, ^10^, ^14^, ^15^, ^16^, ^17^, ^18^, ^19^

In vitro and in vivo studies in asthma indicate that specific and non‐specific provoking agents can induce bronchial remodeling independent of inflammation. Active provocations with allergen (which causes bronchospasm and eosinophilic inflammation) or methacholine (which causes bronchospasm without eosinophilic inflammation) are performed to show the effect of specific and non‐specific factors on molecular underpinnings in patients with asthma.^5^, ^10^, ^14^, ^15^, ^16^, ^17^, ^18^, ^19^

## 2 AIM

The aim of this study was to evaluate the effect of nasal allergen challenge and methacholine challenge test on changes in mRNA expression of genes encoding the TGF‐β (TGF‐β1 and TGF‐β3)–Smad (MPK1/3, Smad1/3/6/7) signaling pathway in peripheral blood mononuclear cell (PBMC) of asthmatic patients.

## 3 MATERIAL AND METHODS

### 3.1 Ethical approval

The study was approved by the local Ethics Committee (consent of the Research Review Board of the Medical University of Lodz, Lodz, Poland; no. RNN/31/14/KE). At the beginning of the study, patients were invited to participate in the study voluntarily and a written informed consent was obtained from every patient prior to the enrolment.

### 3.2 Study group

A convenience sample of in‐ and outpatients with asthma (diagnosed according to the ICD‐10 classification, code J45) was recruited in 2019–2020 from the Department of Internal Medicine, Asthma and Allergy in the N. Barlicki University Clinical Hospital No. 1 of the Medical University of Lodz, the Department of Pneumonology and Allergology in the N. Barlicki University Clinical Hospital No. 1 of the Medical University of Lodz, the Department of General and Oncological Pulmonology in the N. Barlicki University Clinical Hospital No. 1 of the Medical University of Lodz, and the Specialist Outpatient Clinic of Pulmonary Diseases and Allergology in the N. Barlicki University Clinical Hospital No. 1 of the Medical University of Lodz. Medical data for the survey questionnaire (medical questionnaire) were collected by specialists in internal medicine, allergology, and lung diseases. The patients who had been qualified to the study by specialists (given above), underwent intranasal allergen provocations or methacholine tests according to medical recommendations (diagnosis of allergic rhinitis or diagnosis of asthma) and in compliance with the current standards for such tests. Next, 9 ml of blood was collected from patients before the provocation (at the time coded 0 h) and two times after the provocation, at 1 and 24 h. Peripheral venous blood was collected from the ulnar vein. There were two separate patient cohorts in the study. One―patients had a nasal allergen challenge, and the other cohort―methacholine challenge.

### 3.3 Asthma diagnosis

Asthma diagnosis was established according to The Global Initiative For Asthma (GINA) 2019 recommendations, based on clinical asthma symptoms and a lung function test. The level of asthma severity and control was determined on the basis of GINA Report Guidelines. All the participants underwent subjective examinations (including structuralised anamnesis and also an element of subjective examination), also an analysis of factors such as: gender, obesity, tobacco smoking, duration of bronchial asthma, allergy to house dust mites, animal fur, mould spores, cockroaches allergens, hypersensitivity to non‐steroid anti‐inflammatory drugs (NSAIDs), etc., in order to determine their role in the development of resistance to glucocorticoids, as well as to establish whether they are primary or secondary to genetic factors. The detailed information was obtained from medical records of particular patients. If results of spirometry or allergological tests were unavailable, such examinations were additionally performed during the recruitment visit. Subjects suffering from clinically significant exacerbations, using drugs which might have induced resistance to glucocorticoids (such as rifampicin, phenobarbital, phenytoin, effedrine), subjects with signs of viral infections, either generalised, or affecting the respiratory tract, as well as subjects failing to comply with the doctor's recommendations, were excluded from the patient group.^11^, ^13^, ^20^, ^21^, ^22^, ^23^ Spirometry tests were conducted in the Outpatient Department according to the European Respiratory Society (ERS)/American Thoracic Society (ATS) standards, whereas allergological tests were performed according to the European Academy of Allergy and Clinical Immunology (EAACI) guidelines.^11^, ^13^, ^20^, ^21^, ^22^, ^23^

### 3.4 Nasal allergen challenge

The nasal allergen challenge (NAC) was performed in accordance with current EAACI standards and in compliance with the manufacturer's recommendations for the procedures of a provocation with intranasal spray, included in the product specifications (Allergopharma challenge test solutions, Manufacturer: Allergopharma GmbH & Co. KG; Marketing Authorisation Number: 9531). The intranasal provocation test was performed with the use of the “spray” method by administering standardized test solutions of 0.04–0.05 ml, through a spray nozzle supplied by the manufacturer and approved for distribution/use. Test solutions were prepared as follows: 1st provocation: dilution 1:10,000 (or more in highly sensitive patients), 2nd provocation: dilution 1:1000, 3rd provocation: dilution 1:100, 4th provocation: dilution 1:10 and 5th provocation: undiluted test solution. The spray nasal allergen challenge was performed according to the approved for distribution/use protocol No. 9531, which is available on the manufacturer's website.^24^, ^25^, ^26^

### 3.5 Methacholine challenge test

The provocation was performed according to the “ERS technical standard on bronchial challenge testing: general considerations and performance of methacholine challenge tests”. First, the patient performed basic spirometry, and then the patient inhaled, using a dispenser, a gradually increasing amount of the substance causing bronchospasm―methacholine. Forced Expiratory Volume (FEV1) change from baseline was assessed. A reduction in FEV1 of ≥20% was considered significant and the triggering concentration (provocative concentration [PC20]) or the provocative dose (PD20) was determined for this value.^27^

### 3.6 Expression of mRNA by the real‐time RT‐qPCR (real‐time quantitative reverse transcription polymerase chain reaction) technique

Venous blood samples were collected from the participants onto tripotassium ethylenediaminetetraacetic acid (EDTA‐K3; SARSTEDT AG & Co.; Nümbrecht).

10 μg total RNA was extracted from the peripheral blood leukocytes using a RNA extraction reagent (TRI Reagent® Solution, Ambion), according to the standard acid–guanidinium–phenol–chlorophorm method.^28^, ^29^, ^30^ The extracted RNA was analyzed with agarose gel electrophoresis and only cases with preserved 28S, 18S, and 5S ribosomal RNA bands, indicating good RNA quality, were used in the study. The amount of purified RNA was determined using spectrophotometry at 260 nm in a Nanodrop analyser (ND‐100; Nanodrop Technologies). The purity and amount was verified according to the ratio of 260/280 nm measurements, and values between 1.8 and 2.1 indicated that the quality of the obtained RNA was optimal and suitable for the quantitative real‐time polymerase chain reaction (RT‐qPCR).^12^, ^21^, ^30^ Expression analysis of the studied genes was performed in the Laboratory of Personalized Medicine and Biotechnology Laboratory of BioNanoPark, Regional Science and Technology Park in Lodz (93‐465 Lodz, Poland).

Expression was analyzed for the following genes:

mitogen‐activated kinases, which play a role in regulating the response to external signals reaching the cell and influence gene expression, division, differentiation, movement, and apoptosis of cells:
  MAPK1 (Mitogen‐Activated Protein Kinase 1),
  MAPK3 (Mitogen‐Activated Protein Kinase 3),
SMAD family genes, encoding proteins serving as signal transducers and transcription modulators and which mediate many signaling pathways:
  SMAD1 (SMAD family member 1),
  SMAD3 (SMAD family member 3),
  SMAD6 (SMAD family member 6),
  SMAD7 (SMAD family member 7),
transforming growth factor beta TGF‐β:
  TGF‐β1 (transforming growth factor β1―controls cell growth, proliferation, differentiation, and apoptosis);
  TGF‐β3 (transforming growth factor β3).

For the purpose of internal control, the *β*‐2 microglobulin (*β‐2M*) gene was used, which demonstrates expression at a constant level in the tested samples. Appropriate TaqMan probes that do not react with genomic DNA were chosen for the eight genes and the control gene (*β‐2M*), selected for the analysis. They are presented in Table 1.

**TABLE 1:** Analyzed genes and the applied TaqMan probes

Gen	Assay ID
*SMAD1*	Hs00195432_m1
*SMAD3*	Hs00969210_m1
*SMAD6*	Hs00178579_m1
*SMAD7*	Hs00998193_m1
*TGF‐β1*	Hs00998133_m1
*TGF‐ β 3*	Hs01086000_m1
*MAPK1*	Hs01046830_m1
*MAPK3*	Hs00385075_m1
*β‐2M*	Hs00187842_m1

For each sample, threshold cycle (CT) values were calculated with the use of Mx‐Pro software. The RT‐qPCR amplification of each gene was compared to that of *β*‐2M (beta‐2‐mikroglobulina), a house‐keeping reference gene, and ΔCT values were determined (ΔCT = CT, GENE – CT, *β*‐2M). The RT‐qPCR data was automatically calculated with the data analysis module. The results were analyzed according to the 2^(−ΔΔCT) method with assumption of 100% reaction yield. Validation of polymerase chain reaction (PCR) efficiency was performed with a standard curve.^12^, ^13^, ^21^, ^28^, ^29^, ^30^ The complementary DNA (cDNA) was subjected to real‐time quantitative PCR using gene‐specific primers for the studied genes and *β*‐2M with the use of the TaqMan Sonds® & Master Mixes for RT‐qPCR (Biotium, Inc.).

The analysis of expression of the selected genes was performed with a Real‐Time PCR Optical Thermocycler manufactured by Biometra (Biometra Biomedizinische Analytik GmbH) using commercial TaqMan probes. TaqMan probes are hybridization probes, designed to increase the specificity of Real‐Time PCR reactions.

The experiment involved carrying out a preliminary optimization of the reaction conditions followed by checking the expression of the studied genes in all samples. A detailed description of the RT‐qPCR reaction conditions is presented in Table 2.

**TABLE 2:** RT‐qPCR reaction conditions for the analyzed expression of the studied genes

Stage	Temperature (℃)	Time (s)	Number of cycles
UNG incubation	50	120	1
Polymerase activation	95	600	1
Denaturation	95	15	45
Annealing	60	60	45

Abbreviation: UNG, Uracil‐DNA‐Glycosylase.

Assays were performed in two repetitions for each sample. Averaged Ct values for the two replicates make up the results of the study. Ct is the number of amplification cycles of the PCR product in which the fluorescence level of the dye exceeds the threshold called the limit cycle. Considering the Ct value, it is possible to analyze the amount of baseline cDNA for the selected genes in the studied sample, and thereby to analyze their expression.^12^, ^13^, ^21^, ^28^, ^29^, ^30^, ^31^

### 3.7 Data analysis

Analysis of missing data was included in the investigation. Blood sampling in at least two (out of three) time points was sufficient for a patient to be included in the study.

Non‐detects of Ct values in qPCR were imputed based on the conditional expectation calculated in the expectation‐maximization algorithm.^32^ Missing ΔCt values for dropouts were imputed with multiple imputation by chained equations (MICE) under a missing at random assumption about the unobserved data. The main analysis was performed in a fully imputed dataset. Moreover, two sensitivity analyses were carried out: 1) dataset before the MICE procedure, 2) fully imputed dataset with adjustment for potential confounders (perennial allergy, administration of inhaled corticoids in a dose exceeding 1000 μg budesonide a day, administration of systemic steroids within past 3 months, current cigarette smoking).

One‐way repeated measures analysis of variance (ANOVA) was performed in order to assess whether the gene expression changed over time following both the provocations. For evaluation of this effect, minimum size of the patients' sample was estimated to be 43 with an assumption of default medium effect size of Cohen's *f* of 0.25, correlation between repeated measures of 0.5, with power set to 0.95 and correction for non‐sphericity being not included. Two‐way repeated measures ANOVA was performed with two‐way interaction evaluated in order to determine whether the provocation type affected the gene expression over time. In the case of sensitivity analysis 1 (with missing data), a mixed effects model was fitted instead of ANOVA. Sensitivity analysis 2 was made with general linear modeling. The Greenhouse‐Geisser (G‐G) correction was applied to adjust to the potential lack of sphericity in each of the analyses. Sphericity implies equal variability of differences between time‐points of gene expression; if the assumption is violated, *p*‐values may be falsely low; G‐G correction is to proportionally inflate the *p*‐values to make the test results more accurate.

Dimensionality reduction was performed using the explorative factor analysis with varimax raw rotation to further explore interrelation of gene expressions. It was done following the Kaiser–Meyer–Olkin (KMO) measure calculation and Bartlett's test of sphericity and performed according to eigenvalue‐below‐one and scree plot criterion. High KMO values (>0.7) and significant results of the Bartlett's test of sphericity indicate that there is a substantial correlation between the explored variables that may call for reduction of the number of factors.

*p*‐values below 0.05 were considered statistically significant. The analysis was performed using the STATISTICA 13.1 software (StatSoft) and R Software version 3.6.1 (R Core Team 2019) was additionally used for MICE procedure.

## 4 RESULTS

120 asthmatic patients were invited to participate in the study. The inclusion and exclusion criteria were met by 98 patients with asthma. From this patient group, another 9 subjects could not be administered an allergen challenge with intranasal provocation because they did not comply with the grace period for antihistamines, and another 13 subjects could not be administered a methacholine challenge because they failed to comply with the grace period for inhaled drugs. One participant dropped out for family reasons. 75 people were included to the study.

There were 4 (5%) missing patients in time‐point “0 h”, 8 (11%) in time‐point “+1 h” and 56 (75%) in time‐point “+24 h”.

In the RT‐qPCR procedure, the mean raw Ct value for the reference gene was 29.1, and the mean Ct values for the tested genes ranged from 32.5 for TGFβ1 to 42.6 for SMAD6 (Table 3). The number of no‐detects also depended on the gene and ranged from 3 (1.9% for TGFβ1) to 128 (81.5% for SMAD6), with a median value of 29.5 (18.8%).

**TABLE 3:** The mean raw Ct values for the tested genes

Tested gene	*MPK1*	*MPK3*	*SMAD1*	*SMAD3*	*SMAD6*	*SMAD7*	*TGFB1*	*TGFB3*
Mean Ct value	36.5	37.5	38.7	35.7	42.6	38.1	32.5	39.4

*Note*: Number of cycles performed in the polymerase chain reaction was 45.

Abbreviation: MPK, mitogen‐activated protein kinase.

Of the 75 subjects who were included in the study, 27 (36%) were applied an intranasal provocation and 48 (64%) a methacholine provocation. Methacholine provocation led to substantial reduction in FEV1 by 16.9 ± 10.7 (%), *p* < 0.0001 (one‐sample *t*‐test). There were 9 men and 18 women in the intranasal provocation group, and 17 men and 31 women in the methacholine test group. The two subgroups did not differ by gender proportion. Detailed characteristics of both study populations, including demographic and clinical parameters, are presented in Table 4.

**TABLE 4:** Demographic and clinical characteristics of the group of study participants divided into subgroups: subjects with NAC and MET

Variable	Number (frequency) or mean (standard deviation)^a^			*p*‐value for comparison
	Total (*n* = 75)	MET (*n* = 48)	NAC (*n* = 27)	
Sex				
Male	26 (35%)	17 (35%)	9 (33%)	0.86^b^
Female	49 (65%)	31 (65%)	18 (67%)	
Age				
(years)	39.7 (15.6)	43.5 (16.2)	32.8 (11.7)	0.0036^c^
BMI				
(kg/m^2^)	25.4 (4.9)	25.9 (4.9)	24.6 (5.0)	0.27^c^
Allergy				
Seasonal	28 (37%)	10 (21%)	18 (67%)	<0.0001^b^
Perennial	25 (34%) (*n* = 74)	12 (26%) (*n* = 47)	13 (48%)	0.048^b^
Number of allergens:	2.0 (2.6) median (1^st^–3^rd^ quartile): 0 (0–4)	1.4 (2.6) median (1^st^–3^rd^ quartile): 0 (0–2)	3.0 (2.3) median (1^st^–3^rd^ quartile): 3 (0–5)	0.0017^d^
	Range: 0–9 (*n* = 74)	Range: 0–9 (*n* = 47)	Range: 0–7	
Nicotine smoking				
Current smokers	13 (17%)	11 (23%)	2 (7%)	0.12^e^
Has not smoked for at least 6 months; used to smoke	18 (24%)	13 (27%)	5 (19%)	0.40^b^
Number of pack‐years^f^	4.3 (8.5) median (1^st^–3^rd^ quartile): 0 (0–5)	5.1 (9.5) median (1^st^–3^rd^ quartile): 0 (0–6)	3.0 (6.5) median (1^st^–3^rd^ quartile): 0 (0–1)	0.13^d^
	Range: 0–42	Range: 0–42	Range: 0–20	
Rhinitis				
Rhinitis (any type)	53 (71%)	28 (58%)	25 (93%)	0.0018^b^
Rhinitis treated with nasal GCSs	19 (25%)	6 (13%)	13 (48%)	0.0007^b^
Episodic rhinitis	17 (23%)	12 (25%)	5 (19%)	0.52^b^
Chronic rhinitis	38 (51%)	16 (33%)	22 (81%)	<0.0001^b^
Seasonal rhinitis	19 (25%)	12 (25%)	7 (26%)	0.93^b^
Perennial rhinitis	36 (48%)	16 (33%)	20 (74%)	0.0007^b^
Medication use				
anti – H_1_	30 (40%)	16 (33%)	14 (52%)	0.12^b^
PPI	8 (11%)	6 (13%)	2 (7%)	0.70^e^
anti – H_2_	2 (3%)	1 (2%)	1 (4%)	1.00^e^
Intolerance, hypersensitivity to drugs	4 (5%)	3 (6%)	1 (4%)	1.00^e^
Comorbidities				
Nasal polyps current, recurrent, postoperative	3 (4%)	2 (4%)	1 (4%)	1.00^e^
Neurological or neurosurgical diseases	19 (25%)	15 (31%)	4 (15%)	0.12^b^
Lipid disturbances, including hypercholesterolemia	5 (7%)	5 (10%)	0 (0%)	0.15^e^
Goitre	1 (1%)	1 (2%)	0 (0%)	1.00^e^
Hypoactivity	2 (3%)	2 (4%)	0 (0%)	0.53^e^
Hyperactivity	4 (5%)	4 (8%)	0 (0%)	0.29^e^
Atherosclerosis	1 (1%)	1 (2%)	0 (0%)	1.00^e^
Hypertension	10 (13%)	8 (17%)	2 (7%)	0.31^e^
Arrhythmia	5 (7%)	5 (10%)	0 (0%)	0.15^e^
Myocardial infarction	1 (1%)	1 (2%)	0 (0%)	1.00^e^
Other cardiac diseases	2 (3%)	2 (4%)	0 (0%)	0.53^e^
Other pulmonary diseases^g^	1 (1%)	1 (2%)	0 (0%)	1.00^e^
Gastric ulcer	4 (5%)	3 (6%)	1 (4%)	1.00^e^
Duodenal ulcer	1 (1%)	1 (2%)	0 (0%)	1.00^e^
Reflux diseases or suspicion of reflux disease	10 (13%)	8 (17%)	2 (7%)	0.31^e^
Neoplasmatic diseases or medical history of neoplasmatic disease	1 (1%)	1 (2%)	0 (0%)	1.00^e^
Immunodeficiency disorders	3 (4%)	2 (4%)	1 (4%)	1.00^e^
Specific immunotherapy	9 (12%)	3 (6%)	6 (22%)	0.06^e^

*Note*: A detailed description in the article. The author's own design.

Abbreviations: MET, methacholine test; NAC, nasal allergen challenge.

^a^ If not stated otherwise.

^b^ Pearson's χ^2^ test.

^c^ Student's *t*‐test.

^d^ Mann–Whitney *U* test.

^e^ Fisher's exact test.

^f^ Pack‐year—number of packs of cigarettes smoked daily × number of years of smoking.

^g^ Other pulmonary diseases including: sarcoidosis, ectasis, tuberculosis (TBC), chronic obstructive pulmonary disease (COPD).

We found that both the intranasal allergen challenge (specific agents) and methacholine challenge (non‐specific agent) contributed to changes in the relative expression of genes of the TGF‐β (TGF‐β1 and TGF‐β3)‒Smad (MPK1/3, Smad1/3/6/7) signaling pathway in peripheral blood leukocytes of asthmatic patients. An analysis of the relative gene expression changes showed changes for *TGF‐β1, MAPK1, MAPK3, Smad3, Smad6* and *Smad7* genes. There were no significant changes in the expression of TGF‐β3 and Smad1 genes over time after the provocations. Detailed results are presented in Table 5.

**TABLE 5:** mRNA gene expression changes over time following provocations; relative gene expression was calculated as a ratio of gene expression in +24 h time‐point versus 0 h time‐point

		MAPK1	MAPK3	SMAD1	SMAD3	SMAD6	SMAD7	TGF‐β1	TGF‐β3
Effect size	Relative gene expression (95% CI)	0.71 (0.53–0.96)	0.60 (0.45–0.80)	0.63 (0.41–0.98)	0.58 (0.42–0.80)	0.40 (0.24–0.66)	0.39 (0.21–0.72)	2.27 (1.40–3.69)	0.84 (0.47–1.50)
	Partial *η* ^2^	4.65%	8.58%	3.47%	8.16%	8.36%	7.72%	10.21%	0.69%
*p*‐value in main analysis		0.038	0.0014	0.078	0.0021	0.0017	0.0054	0.0007	0.58
*p*‐value in sensitivity analysis 1		0.20	0.037	0.066	0.11	0.013	0.0026	0.18	0.097
*p*‐value in sensitivity analysis 2		0.13	0.034	0.12	0.021	0.046	0.021	0.0026	0.091

*Note*: The analysis was performed for the total sample of allergen and methacholine provocation patients. Details are included in the article.

A data analysis was also performed in order to determine whether the provocation type affected the gene expression over time. However, we did not analyze the influence of specific provocation on the change in gene expression, as shown in Table 6.

**TABLE 6:** Difference in mRNA gene expression changes over time following intranasal and methacholine provocations

Variable	MPK1	MPK3	SMAD1	SMAD3	SMAD6	SMAD7	TGFB1	TGFB3
Partial *η* ^2^	0.28%	1.41%	0.90%	2.01%	1.69%	2.36%	4.14%	0.63%
*p*‐value in main analysis	0.77	0.35	0.50	0.23	0.29	0.18	0.053	0.61
*p*‐value in sensitivity analysis 1	0.70	0.67	0.49	0.85	0.66	0.15	0.88	0.40
*p*‐value in sensitivity analysis 2	0.64	0.070	0.33	0.11	0.18	0.35	0.20	0.90
*p*‐value in sensitivity analysis 3	0.69	0.072	0.51	0.37	0.53	0.45	0.43	0.87

*Note*: Sensitivity analysis 3 was added to account for between‐group baseline data difference in age and number of allergens.

Abbreviation: MPK, mitogen‐activated protein kinase.

**FIGURE 1:**
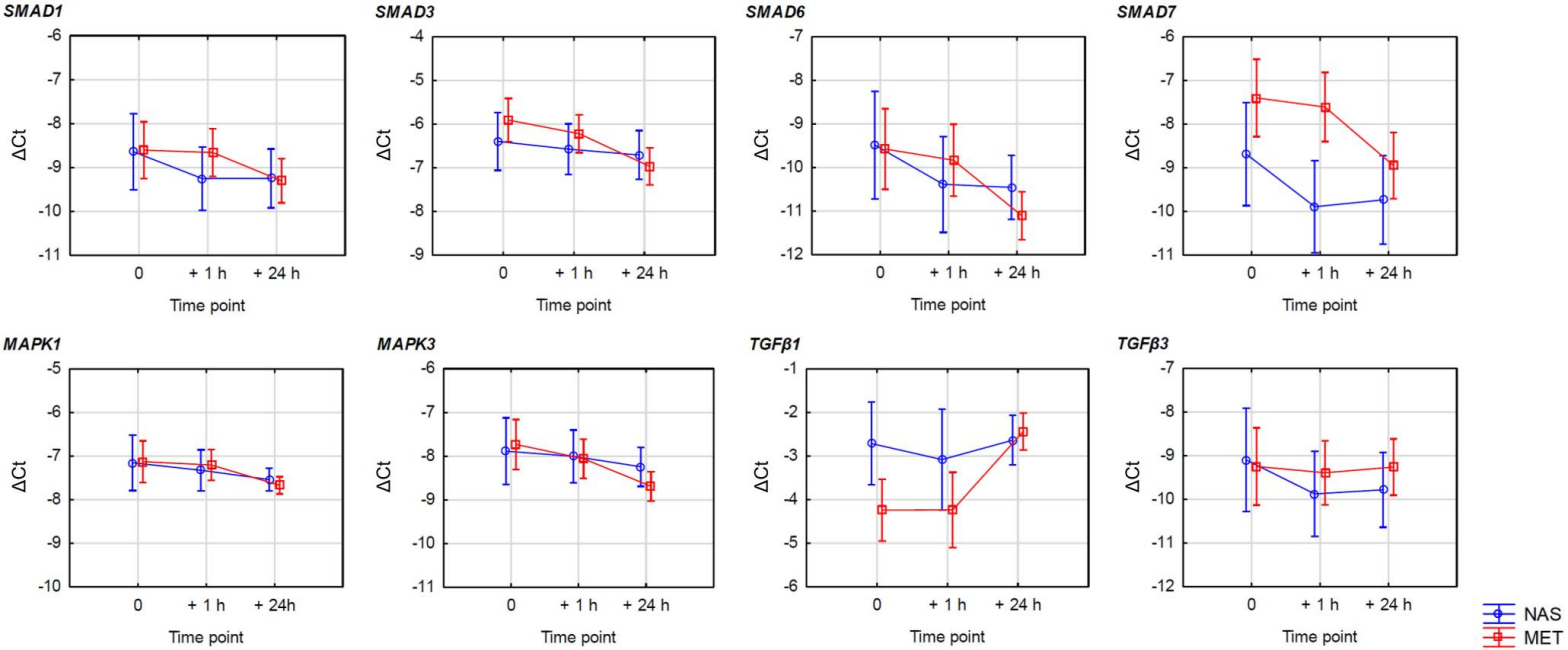
mRNA gene expression following provocation over time. Gene expression estimates are expressed as mean −ΔCt values (decrease in the value by a unit means a 2‐fold decrease in gene expression) with the whiskers indicating 95% confidence intervals. MET, methacholine provocation; NAS, intranasal provocation

Figure 1 provides a graphical illustration of the analysis of changes in the expression of TGF‐β (TGF‐β1 and TGF‐β3)–Smad (MPK1/3, Smad1/3/6/7) signaling pathway genes in asthmatic patients by the type of provocation. Despite relatively small changes in the expression, a similar downward trend in the relative expression of Smad (MPK1/3, Smad1/3/6/7) was noted, irrespective of the type of performed intranasal allergen or methacholine challenges (except for increase in TGF‐β1and non‐significant effect on TGF‐β3). The details of the observed relationships are visualized as mean −ΔCt values, as detailed in Figure 1.

**FIGURE 2:**
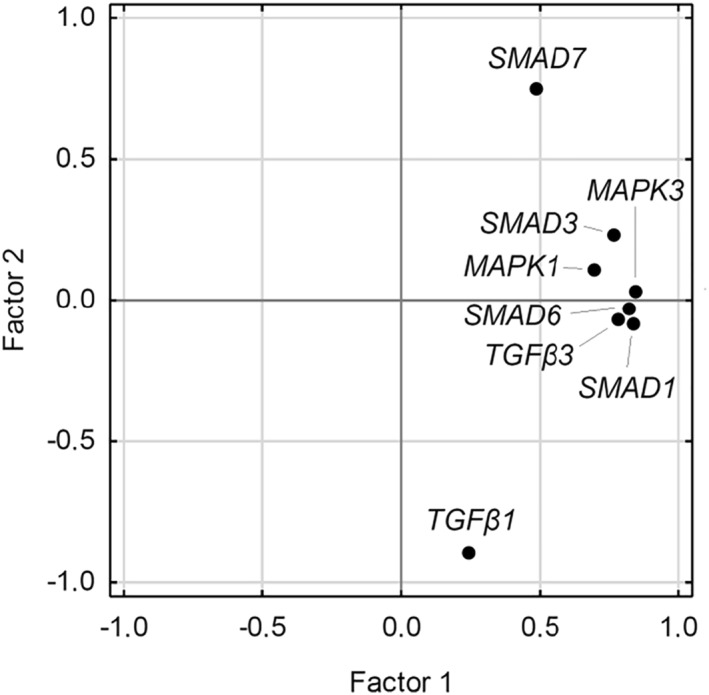
Interrelation of mRNA gene expressions in two‐dimensional representation. Factor 1 retains 51.5% of variance, whereas Factor 2–17.6%. Kaiser–Meyer–Olkin measure = 0.768, the Bartlett's test of sphericity: χ^2^(28) = 960.0, *p* < 0.0001. The analysis was performed in a fully imputed dataset

This similar trend in gene expression over time regardless of the provocation type urged to explore the interrelation of the analyzed genes' expression. The KMO measure and the Bartlett's test of sphericity provided adequate rationale for this. Reduced dimensionality of gene expression to two factors allowed to retain as much as almost 70% of variance, as shown in Figure 2, which indicates high intercorrelation of the expression of tested genes, particularly SMAD1/3/6, MAPK1/3, and TGFβ3 with each other as well as negative correlation of SMAD7 with TGFβ1.

## 5 DISCUSSION

This study reveals that extrinsic factors, both specific, such as: allergens, and non‐specific, such as: methacholine, have a significant effect on changes in the mRNA expression of key genes of the TGF‐β (TGF‐β1)–Smad (MPK1/3, Smad3/6/7) signaling pathway in peripheral blood leukocytes of asthmatic patients.

First of all, we point out that the used provocative factors increased the expression of the cytokine TGF‐β1 alone. This was not observed for TGF‐β3. This is important in asthmatic patients because isoform 1 strongly induces macrophage and fibroblast chemotaxis, stimulates fibroblast proliferation and synthesis, stimulates synthesis of fibronectin, proteoglycans, collagen type I and III, enhances eosinophil chemotaxis after allergen exposure, and causes phosphorylation of MAPK kinases―increased bronchial myocyte proliferation. Thus, it is the strongest known inducer of bronchial remodeling in asthmatic patients, as it was confirmed by previous reports.^5^, ^11^, ^12^, ^14^, ^20^, ^21^ It should be noted, however, that inconsistent results regarding the difference in TGF‐beta1 expression change between two provocation types were obtained, and the matter of effect of particular provocation is inconclusive. We did not study them separately because there were too few patients in the database to conduct such an analysis.

Interestingly, we noticed an increase in the relative gene expression for MAPK kinase isoforms 1 and 3, after both types of provocation over time. This is an important observation because TGF‐β1, through the TGFβRI/TGFβRII receptor (ALK5) and MAPK1/3 kinases, independent of Smad signaling proteins, inhibits collagenase and matrix metalloproteinases gene expression, inhibits MHC type II antigen expression and surfactant synthesis by type II pneumocytes. This changing in the expression of these molecules may significantly reduce the progress of bronchial remodeling.^5^, ^11^, ^12^, ^14^, ^20^, ^21^

Analyses of the Smad1 gene expression did not show its significant change after performing an allergen and a methacholine challenge test and exposing the asthmatic patient to either a specific or non‐specific factor. However, the results were close to the borderline of statistical significance. Consequently, the results of Smad1 mRNA expression should be considered inconclusive. Therefore, both possibilities should be considered: the change in Smad1 protein expression may be dependent or independent of the ongoing underlying inflammation factors in asthma. If Smad1 expression is related to provocation, this can probably be explained in many ways. In our opinion, it may be due to the fact that Smad1 receives its signal from a different type of ALK receptor than Smad signal proteins 3, 6, and 7. Indeed, ALK1, 2, 3, and 6 receptors are the main signal transducers from TGF‐β1 to Smad1, and not from ALK5. The Smad1 signaling protein may have a different function in response to specific and non‐specific provocations than the other studied Smad proteins.^5^, ^10^, ^17^, ^18^, ^19^ We conclude this because of high correlation of Smad1 with other tested genes. However, borderline significance allows for speculative discussion only.

Interesting is the change in the Smad3 mRNA expression after the provocation triggered by irritants in allergen and methacholine provocations. A significant change in the relative expression of the Smad3 gene is known to correlate with activation of the Smad2/3 complex and stimulates the intranuclear Smad2/3/4 protein systems and TF, leading to the activation of target gene transcription, including those responsible for bronchial remodeling in asthma, particularly those of MMPs, PAI‐1, CTGF, MCP‐1, IL‐6, TGF‐β, TSP‐1, TGFR‐1/2, fibronectin, proteoglycans, as well as type I and III collagen.^5^, ^33^, ^34^, ^35^

In the above context, the role of Smad6 and Smad7 proteins, which belong to the group of inhibitory proteins (I‐Smad) for the TGF‐β ‐ Smad signaling pathway, is interesting and they respond to signals transmitted by ALK1 and ALK5 receptors. Their role in asthma has not been fully understood.^5^, ^10^, ^18^, ^36^, ^37^, ^38^

In this study, we also performed an exploratory factor analysis of the relative expression change of the studied mRNA genes of the TGF‐β–Smad signaling pathway after exposure to provocative factors, as shown in Figure 2. Substantial interrelation of the expressions is a very interesting observation. It shows that there are two factors that determine the cluster change of expression of groups of studied genes of the *TGF‐β* (*TGF‐β1* and *TGF‐β3*)–*Smad* (*MPK1/3, Smad1/3/6/7*) signaling pathway in asthmatic patients. The first leading factor correlates with the majority of genes (except for *Smad7* and *TGF‐β1*). The other one is independent of most of these genes, however, it relates to *Smad7* and *TGF‐β1* in an opposing way. This interrelation also can be observed by analyzing similar trend lines for the studied gene expression changes as illustrated in Figure 1.

The point is that the factor analysis shows a high correlation of “the expression” of certain genes, but not “changes in the expression” of genes. Thus, we cannot claim that a decrease in one of *TGF‐β3, MPK1/3,* and *Smad1/3/6* genes after a provocation entails a decrease of the other genes. Results of the factor analysis should be probably interpreted like this: if the expression of one of these genes at any time point (before or after a provocation) in a particular patient is high, the other genes also demonstrate high expression. Thus, the genes co‐work and their expression seems to “coordinate”.

These interrelations can be explained by interplay of the tested gene products called signal transduction and thus their expression may occur in concert. This observation is likely the curious reported in scientific literature and may contribute to deeper understanding of the molecular pathophysiology of asthma. TGF‐β1 cytokine is an important factor responding to external irritants in asthma, leading to the activation of the two most important receptor groups ALK1 and ALK5, which in turn are responsible for further activation of the entire Smad and MAPK pathways.

To make the analysis more reliable, we wanted to add that sensitivity analyses to support the robustness of the obtained results. The observations were described using statistical methods with all appropriate statistical corrections. This fact was included in the Materials and Methods section.

## 6 LIMITATIONS

Observations were made on 75 participants who underwent an intranasal allergen and bronchial methacholine challenge for diagnostic clinical indications, and who, were different in terms of age and the number of allergens (see Table 4). It is a naturalistic observational study, and patients were allocated to groups based on diagnostic and therapeutic indications, but not randomly. Blood was collected from the patients only at three time points: 0 h, 1 h, and 24 h. The patients' blood was used to perform the tests, not any of their tissue material. Hence, there are no different biological materials that could potentially show differences in the expression of the studied genes that could be used for molecular comparative studies.

Besides, the patients were not prevented from the impact of external environmental factors for a sufficiently long time before specific and non‐specific tests were performed. The modifying effect on the results of the experiment could possibly have had many elements. They included potential confounding factors, such as medications taken by patients, disease duration, comorbidities, duration of the challenge itself, analysis carried out at only two time points (0 h, 1 h, and 24 h), smoking, mutations and polymorphic forms tested by genes, the level of oxidative stress and free radicals, and many other environmental factors. Here, we wanted to show some trends and relationships rather than describe the strictly isolated biochemical and molecular reactions.

The role of BMP proteins, which can modify the expression of Smad1/5 and Smad4 by interacting with ALK1,2,3,6 receptors and interfering with the mRNA expression of the studied genes, was not taken into account either, which had been assumed to be included in this research project. Multicentre experiments on comparable in vitro, animal, and in vivo models, including several blood collections in patients at different time intervals after activation of standardized doses of specific and non‐specific irritants would be a valuable addition to our work.

Apart from the limitations presented in the manuscript by the authors above, gene expression was examined without reference to their protein products and it is not always the level of gene expression corresponds to/correlates with the amount of protein product. Further research is needed at the protein level.

## 7 CONCLUSION

Our experiment allows us to conclude that both examined types of challenges contributed to changes in the relative expression of majority of genes of the *TGF‐β* (*TGF‐β1* and *TGF‐β3*)–*Smad* (*MPK1/3, Smad1/3/6/7*) signaling pathway in asthmatic patients. A significant change in the mRNA expression of the *TGF‐β1‐ MPK1/3,* and *Smad3/6/7* genes occurred after an intranasal allergen and bronchial methacholine challenge. TGF‐β1 expression increased after methacholine and allergen provocation, which is the main factor activating the entire signaling pathway in asthma, which, we believe, may be clinically useful. Activation of ALK1 and 5 receptors by TGF‐β1 is followed by stimulation of Smad‐independent and MAPK (Smad‐independent) proteins, which is a leading factor responsible for bronchial remodeling in asthma. The discovered change in the mRNA I‐Smad expression in asthma still remains unknown and requires further scientific studies. The cluster change in the expression of groups of TGF‐β–Smad signaling pathway genes, studied in asthmatic patients (cluster 1: TGF‐β1 and Smad7, and cluster 2: TGF‐β3, MPK1/3, Smad1/3/6), may be a reason for clustering genetic elements into asthma phenotypes and conducting a deeper analysis of the similarity of interactions between different groups of proteins involved in the chronic inflammatory process. The changes in the expression of the studied genes observed by us are probably an adaptive reaction to the stimulation of signaling in the TGF‐β/Smad and MPK pathway.

To sum up, the TGF‐β‐Smad signaling pathway is an important element modeling bronchial inflammation in asthma. The change in the mRNA expression of the *TGF‐β1‐ MPK1/3,* and *Smad3/6/7* genes occurs after an intranasal allergen and bronchial methacholine challenge. These findings have potential implications for asthma treatment.

## AUTHOR CONTRIBUTIONS

**Michał Gabriel Panek**: Conceptualization (equal); Data curation (equal); Formal analysis (equal); Funding acquisition (equal); Investigation (equal); Methodology (equal); Project administration (equal); Resources (equal); Software (equal); Supervision (equal); Validation (equal); Visualization (equal); Writing – original draft (equal); Writing – review & editing (equal). **Michał Seweryn Karbownik**: Formal analysis (equal); Software (equal); Validation (equal); Visualization (equal); Writing – review & editing (equal). **Karol Maksymilian Górski**: Formal analysis (equal); Methodology (equal). **Marcelina Koćwin**: Data curation (equal); Resources (equal). **Grzegorz Kardas**: Data curation (equal); Resources (equal); Supervision (equal). **Mateusz Marynowski**: Software (equal). Writing – review & editing (equal). **Piotr Kuna**: Writing – review & editing (equal).

## CONFLICT OF INTEREST

The authors declare that the research was conducted in the absence of any commercial or financial relationships that could be construed as a potential conflict of interest.
